# Learning-based control approaches for service robots on cloth manipulation and dressing assistance: a comprehensive review

**DOI:** 10.1186/s12984-022-01078-4

**Published:** 2022-11-03

**Authors:** Olivia Nocentini, Jaeseok Kim, Zain Muhammad Bashir, Filippo Cavallo

**Affiliations:** 1 Department of Industrial Engineering, University of Florence, Florence, Italy; 2 The BioRobotics Institute, Scuola Superiore Sant’Anna, Pontedera, PI Italy; 3 Department of Excellence in Robotics and AI, Scuola Superiore Sant’Anna, Pisa, Italy

**Keywords:** Service robots, Robotic dressing assistance, Manipulation of clothes, Human–robot interaction

## Abstract

**Background:**

Service robots are defined as reprogrammable, sensor-based mechatronic devices that perform useful services in an autonomous or semi-autonomous way to human activities in an everyday environment. As the number of elderly people grows, service robots, which can operate complex tasks like dressing tasks for disabled people, are being demanded increasingly. Consequently, there is a growing interest in studying dressing tasks, such as putting on a t-shirt, a hat, or shoes. Service robots or robot manipulators have been developed to accomplish these tasks using several control approaches. The robots used in this kind of application are usually bimanual manipulator (i.e. Baxter robot) or single manipulators (i.e. Ur5 robot). These arms are usually used for recognizing clothes and then folding them or putting an item on the arm or on the head of a person.

**Methods:**

This work provides a comprehensive review of the most relevant attempts/works of robotic dressing assistance with a focus on the control methodology used for dressing tasks. Three main areas of control methods for dressing tasks are proposed: Supervised Learning (SL), Learning from Demonstration (LfD), and Reinforcement Learning (RL). There are also other methods that cannot be classified into these three areas and hence they have been placed in the other methods section. This research was conducted within three databases: Scopus, Web of Science, and Google Scholar. Accurate exclusion criteria were applied to screen the 2594 articles found (at the end 39 articles were selected). For each work, an evaluation of the model is made.

**Conclusion:**

Current research in cloth manipulation and dressing assistance focuses on learning-based robot control approach. Inferring the cloth state is integral to learning the manipulation and current research uses principles of Computer Vision to address the issue. This makes the larger problem of control robot based on learning data-intensive; therefore, a pressing need for standardized datasets representing different cloth shapes, types, materials, and human demonstrations (for LfD) exists. Simultaneously, efficient simulation capabilities, which closely model the deformation of clothes, are required to bridge the reality gap between the real-world and virtual environments for deploying the RL trial and error paradigm. Such powerful simulators are also vital to collect valuable data to train SL and LfD algorithms that will help reduce human workload.

## Introduction

Over the latest years, there has been an increasing interest in Human Robot Interaction (HRI) due to the increasing usage of robots not only in industries, but in other areas such as schools [1], homes [2], hospitals [3], and rehabilitation centres [4]. Service robotics is one such area of robotics where robots have shown high promise in working near humans. Intelligent robotic agents have been deployed in hospitals [5], in domestic environments [6], in retirement houses [7]. The presence of a robot, in fact, is a useful support during the management of daily activities [8, 9], the promotion of social inclusion [10], and the suggestion of healthy activities [11, 12]. An easy and continuous connection with other people (i.e. relatives, friends or doctors), could promote social inclusion of people with disabilities or elderly people and increase the quality of their life [13]. Consequently, in the future, robots will concretely share environments with human beings to actively collaborate with them in specific daily tasks.

One such daily task is the handling of clothes, ranging from washing them to pressing and folding them, or to placing them at their designated places such as cupboards and shelves. Getting dressed up is another daily task involving the handling of clothes. While accomplishing these tasks may seem to be an effortless task for the young and able-bodied, it is undoubtedly a cumbersome activity for the elderly and the disabled and thus demands assistance. The increased life expectancy, owing to the availability of better healthcare facilities, coupled with falling fertility levels [14] has only added to the already ageing population. This has directly resulted in a shortage of caregivers and therapists [15]. This shortage has prompted researchers in the field of robotics to explore new avenues and ways of letting robots take over, fully or partially, some of the assistive tasks involving cloth manipulation.

These manipulation tasks, as trivial as they may seem for humans, are extremely challenging for robots to accomplish. These challenges stem from the intrinsic property of clothes being deformable which allows them to, in theory, assume an infinite number of states each varying in appearance. The challenge is aggravated by the unpredictability of the outcome of a specific action on a piece of cloth. Thus, tracking the cloth state becomes an expensive operation once a manipulation action is executed. On top of that, effective trajectory planning, and control strategies are needed to execute such manipulation in a closed loop which remains a challenge too. In fact, an intelligent robotic agent requires a perfect synergy between the state perception and control execution to ensure successful completion.

While the state estimation problem has been thoroughly studied over the recent years, there still exists a gap in identifying ideal closed loop control strategies for different cloth manipulation scenarios. Earlier work on state perception started off by employing sophisticated motion detection systems which soon gave way to the use of inexpensive depth cameras and principles of computer vision to infer the cloth state. Many of the works also attempted to model the deformation of clothes to predict their future state. A comprehensive review outlining these methods was carried out by [16]. There has been works aimed at modelling the manipulator movements to achieve cloth manipulation tasks such as folding or assisted dressing, but they have proved to be insufficient in adapting to the complex nature of movements required mainly due to their limited generalization abilities and higher computations costs for working in real-time scenarios. Consequently, researchers have turned to make the robots learn these movements as opposed to modelling them.

Some notable works have employed the Reinforcement Learning paradigm to make robots learn the required trajectories based on trial and error, interacting with the environment (clothes) while others have tried to exploit human demonstrations in a Learning from Demonstration paradigm to impart human knowledge to robotic agents. There exists, however, a pressing need to analyse where the field currently stands. Concretely, the following questions need to be addressed:

1. What is the current state of the art for learning based approaches in cloth manipulation?
2. How effective are these approaches in overcoming the limitations of traditional model-based approaches?
3. What are the current challenges and future directions of research in this area?

To answer these questions, we have carried out this comprehensive review. Our work analyses the learning-based strategies, keeping in mind the above-mentioned questions, and groups them as follows:

- Supervised learning (SL): is the task of learning a function that maps an input to an output based on example input–output pairs [17, 18].
- Learning from demonstration (LfD): is to transfer new skills to a machine based on human demonstrations [19, 20].
- Reinforcement learning (RL): is an area of machine learning concerned with how software agents ought to take actions in an environment so as to maximize some notion of cumulative reward. Its focus is finding a balance between exploration (of uncharted territory) and exploitation (of current knowledge) [21, 22].

The rest of the article is organized as follows: in Sect. 2, the research methodology for the comprehensive review is explained. In Sects. 3 and 4 the results and the discussions regarding the papers are shown. In Sect. 5, a summary of the review and its conclusions are presented.

## Methodology: search strategy and selection criteria

This paper reviews empirical studies published between 2009 and 2019 since most of the advances in this area have occurred within that timeframe. A bibliography was developed upon searches in Scopus, Web of Science, and Google Scholar electronic databases.

The searched terms and the combinations used are: {dress AND robot} OR {garment AND robot} OR {dress AND manipulation} OR {garment AND manipulation} OR {dress AND grasping} OR {garment AND grasping} AND D.

$$D\in \left\{ReinforcemenLearning,Learningbydemonstration,Supervisedlearning\right\}$$

**Fig. 1:**
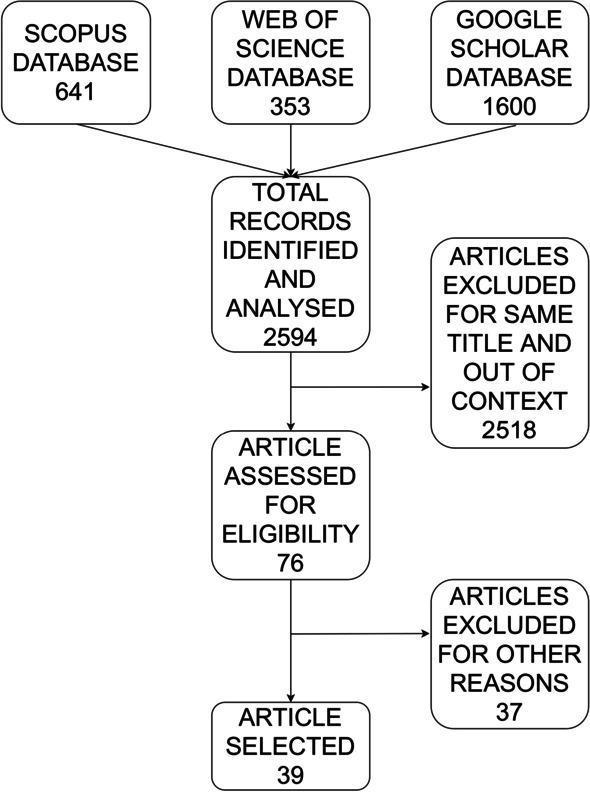
Selection process of relevant papers

As concerns Google Scholar, we took into consideration the first ten pages of the electronic database. Reference lists of included articles and significant review papers were examined by authors of the review to include other relevant studies. After the deletion of duplicates and papers out of context (i.e. papers not related to robots), we identified articles deserving a full review. Additionally, other articles were excluded (not written in English for e.g.) and a total of 76 works was selected at this stage. Then, a full-text assessment was carried, and the final list of papers includes 39 studies, since papers not related to control strategies were discarded; in the following figure (Fig. 1) the selection process is shown:

## Results

### Application overview

The interest toward service robots which are involved in dressing task has grown and we decided to collect the papers concerning dressing dividing them according to the task the robot is doing. Particularly, out of the fully selected papers, 8 papers (20.51%) were published before 2015 and 31 papers (79.49%) were published within the past five years (Table 1).

**Table 1:** List of papers

Refs.	Aim	Task	Robot	Sensors used	Control strategy	Accuracy	Method
Lui al. [25]	Manipulating deformable objects./The robot untangles various ropes	Cloth folding or untangling or coveraging	PR2	Stereo camera pairs Mono-cameras pressure sensor arrays	SL	76.7% for intersection graph inference and 89.2% for node inference	Max-margin learning
Bersch et al. [24]	Transforming an item from a random crumpled configuration into a folded state./A PR2 robot folds a towel	Cloth folding or untangling or coveraging	PR2 robot	Stereo camera pairs Mono-cameras pressure sensor arrays	SL	90%	SVM
Corona et al. [28]	The approach of this paper uses a hierarchy of three CNNs./Grasp a garment using a robot	Cloth folding or untangling or coveraging	Wam robot	Kinect	SL	96.85%	CNNs
Yang et al. [23]	A machine-learning-based humanoid robot that can work as a production line worker and fold clothes./A dual arm robot folds a cloth	Cloth folding or untangling or coveraging	Nextage Open Robot	A camera image whose resolution is 112 × 112 × 3chs (37,632 dimensions, RGB)	SL	Trained Cloth Grabbed 88.9% Grabbed + Folded 88.9% Untrained Cloth Grabbed 77.8% Grabbed + Folded 66.7% Total Grabbed 83.3% Grabbed + Folded 77.8%	DL
Hu et al. [26]	A general approach to automatically visual servo-control the position and shape of a deformable object/Manipulation of a garment by a dual arm robot	Cloth folding or untangling or coveraging	ABB robot	Two 3D cameras	SL	70% for the peg-in-hole task and 90% for other tasks	Fast Online Gaussian Process Regression
Tanaka et al. [14]	The paper presents a motion planning method for automatic operation of cloth products using a robot./The robot folds a garment	Cloth folding or untangling or coveraging	HIRO robot	Kinect	SL	–	NN
Jia et al. [27]	An approach for manipulating high-DOF deformable objects using a random-forest-based controller./A dual-armed robot and a human is holding four corners of a cloth	Cloth folding or untangling or coveraging	ABB YuMi	Realsense camera	SL	–	Random-Forest-Based Imitation Learning
Sannapaneni [29]	Teaching a manipulator using LfD technique to fold clothes./The ADAM robot folds a cloth	Cloth folding or untangling or coveraging	Amrita Dual Anthropo-morphic Manipulator	Logitech camera	LfD	–	Dynamic Movement Primitives (DMP), HMM
Wu et al. [35]	A problem of deformable object manipulation through model-free visual RL./A PR2 manipulating clothes and ropes	Cloth folding or untangling or coveraging	PR2	Kinect	RL	–	Used Maximal Value under Placing (MVP) to select the pick point that has the maximum value
Balaguer et al. [30]	A learning algorithm that combines imitation and RL to perform towel folding tasks./A bimanual manipulator folds towels	Cloth folding or untangling or coveraging	Robotic manipulator	Motion capture system	RL	–	Control policy PoWER
Yaqiang et al. [33]	Describing folding behaviour acquisition of a shirt by a dual-arm robot./The dual arm robot folds a t- shirt	Cloth folding or untangling or coveraging	Baxter	Kinect	RL	–	PILCO algorithm
Balaguer et al. [30]	A learning algorithm is proposed that combines imitation and RL./A dual arm robot folds a garment	Cloth folding or untangling or coveraging	Barrett Arms	Kinect	RL	81.66%	
Koganti et al. (2015)	The authors propose the offline learning of a cloth dynamics model./A dual arm robot puts a shirt on a mannequin	Putting a cloth on user’s arm	WAM	RealSense camera	SL	–	Gaussian Process Latent Variable Model
Zhang et al. (2017)	This paper uses a hierarchical multi-task control strategy to automatically adapt the robot motion and minimize the force applied between the user and the robot caused by user movements. Moreover, there is the online update of the dressing trajectory based on the user movement limitations modelled with the Gaussian Process Latent Variable Model in a latent space, and the density information extracted from such latent space./A dual arm robot puts on a shirt on a person’s arm	Putting a cloth on user’s arm	Baxter	Kinect	SL	–	Gaussian Process Latent Variable Model in a latent space
Chance et al. [37]	A robot was used to dress a jacket onto a mannequin and human participants considering several combinations of user pose and clothing type, while recording dynamic data from the robot, a load cell, and an IMU./Putting a jacket on a mannequin	Putting a cloth on user’s arm	Baxter	Kinect	SL	–	SVM
Stria et al. [38]	A classification of garment categories and a focus particularly on garments being held in a hanging state by a robotic arm./The dual arm puts a shirt on the person’s arm	Putting a cloth on user’s arm	–	ASUS Xtion	SL	82%	SVM
Erickson et al. [39]	A multidimensional capacitive sensing technique that estimates the local pose of a human limb in real time./A PR2 robot pulls a hospital gown onto a participant’s arm	Putting a cloth on user’s arm	PR2	Stereo camera pairs Mono-cameras pressure sensor arrays	SL	–	Fully CNN
Gao et al. [40]	An end-to-end approach for home-environment assistive humanoid robots to provide personalized assistance through a dressing/A Baxter robot assists two users to wear a sleeveless jacket	Putting a cloth on user’s arm	Baxter	Kinect	SL	–	Unsupervised expectation-minimization (EM) algorithm is used to learn Gaussian mixture models (GMMs)
Kapusta et al. [43]	A data-driven haptic perception can be used to infer relationships between clothing and the human body during robot-assisted dressing./The robot puts a hospital gown on the patient's arm	Putting a cloth on user’s arm	Festo linear actuator driven by an Animatics Smart Motor	Force-torque sensor	LfD	–	Hidden Markov models (HMM)
Pignat et al. [41]	A programming by demonstration method to efficiently learn how to dress a person./The dual arm robot puts a jacket on a person’ s arm	Putting a cloth on user’s arm	Baxter Robot	Kinect Sensor motors	LfD	–	Hidden semi-Markov model (HSMM)
Clegg et al. (2018)	Incorporate cloth simulation in the deep RL framework to learn a robust dressing control policy./A simulation of a virtual human wearing a hospital gown	Putting a cloth on user’s arm	–	Haptic sensors	Deep RL	–	Trust Region Policy Optimization (TRPO)
Clegg et al. [71]	The application of haptic aware feedback control and deep RL to robot assisted dressing in simulation./A simulation of putting a hospital gown on a person’s arm	Putting a cloth on user’s arm	Simulated Kuka	Haptic, capacitive, force/torque sensor	RL	100% success rate of the right arm, but a 96% success rate of the left arm	Markov Decision Process (POMDP)
Erickson et al. [39]	A deep recurrent model that, when given a proposed action by the robot, predicts the forces a garment will apply to a person’s body./A PR2 pulls a hospital gown onto a participant’s arm	Putting a cloth on user’s arm	PR2	Haptic, force/torque sensor Kinect	Other methods	–	Deep Haptic Model Predictive Control
Chance et al. [47]	A dressing task using a compliant robotic arm on a mannequin./Getting the arm into the sleeve of the jacket	Putting a cloth on user’s arm	Baxter	Force, wireless accelerometer, gyroscope and joint torque sensors	Other methods	–	No learning methods
Koganti et al. [49]	Aa data-efficient representation to encode task-specific motor-skills of the robot using Bayesian nonparametric latent variable models./The dual arm puts a shirt on the user	Putting a cloth on user’s head	Baxter	Kinect	SL	–	Gaussian Process Latent Variable Model
Saxena et al. [50]	A Deep Learning framework for garment recognition and grasping point detection./Putting a t-shirt on a person	Putting a cloth on user’s head	Baxter	Kinect	SL	Sleeveless T-shirt 96% and 100% of the times in Single View and Multi-view, Full-Sleeved T-shirt 90% and 94% of the times in Single and Multi-View. In the case of Unseen Clothes, Sleeveless T-shirt 76% and 96% of the times in Single and Multi-View, Full-Sleeved T-shirt 40% and 72% of the time in Single and Multi-View	DL
Joshi et al. (2019)	A framework for robotic clothing assistance by imitation learning from a human demonstration to a compliant dual-arm robot./The dual arm robot puts a shirt on the user's head	Putting a cloth on user’s head	Baxter	Kinect	LfD	–	–
Umali et al. (2017)	The authors segment the doffing procedure into a sequence of human–robot actions such that the robot only assists when necessary and the human performs the more intricate parts of the procedure./Robot-Assisted doffing using transfer motions	Putting a cloth on user’s head	Baxter	Two webcams Audio-visual camera Kinect	LfD	–	TrajOpt method
Matsubara et al. (2013)	A novel RL framework for learning motor skills that interacts with non-rigid materials./The robot manipulates a shirt	Putting a cloth on user’s head	WAM	Two stereo cameras	RL	90%	Reward function design with topology coordinates
Koganti et al. [52]	A novel method for the real-time estimation of Human-Cloth relationship./The robot puts on a shirt on a mannequin head	Putting a cloth on user’s head	WAM robot	A Senz3D time of flight (ToF) sensor	RL	–	–
Twardon et al. [53]	The authors consider a robot that learns to put a knit cap on a styrofoam head/A dual arm robot puts a cap on a mannequin’s head	Putting a cloth on user’s head	Mitsubishi PA-10 arms	Kinect	RL	–	Direct policy with gradient free policy
Tamei et al. [54]	This study uses RL to perform the task of clothing assistance where the robot learns to put a mannequin head in a shirt./Putting a shirt into a mannequin	Putting a cloth on user’s head	WAM robot	A Senz3D time of flight (ToF) sensor	RL	–	Policy gradient approach
Shinohara et al. [56]	A novel learning framework for learning motor skills interacting with non-rigid materials by RL./Robot is putting a shirt on user heads	Putting a cloth on user’s head	WAM robot	A Senz3D time of flight (ToF) sensor	RL	90%	–
Klee et al. [57]	An approach for a robot to provide personalized assistance for dressing a user./The manipulator successfully puts a hat on the user	Putting a cloth on user’s head	Baxter robot	Kinect	Other methods	–	Sampling-Based Motion Planning
Canal et al. [58]	A method to perform behaviour adaptation to the user preferences, using symbolic task planning./A manipulator puts a shoe on user’s feet	Putting a cloth on user’s feet	WAM Arm	Kinect	LfD	–	MDP
Yamazaki et al. [59]	An autonomous robot’s method of dressing a subject in clothing with the target task of dressing a person in the sitting pose./The humanoid robot puts trousers on the user	Putting a cloth on user’s leg	Humanoid robot	Xtion sensor, tactile and force sensors	Other methods	73%	–
Lee et al. [5]	A method for learning force-based manipulation skills from demonstrations./The robot ties a knot, folds a towel, erases a whiteboard, and ties a rope to a pipe	Multiple tasks	PR2 robot	Stereo camera Mono-camera pressure sensor arrays	LfD	–	Dynamic Movement Primitives (DMP), HMM, Point cloud
Tsurumine et al. [60]	Deep learning method applied to two real robotic manipulation tasks./The robot has to flip a handkerchief and fold a t-shirt	Multiple tasks	NEXTAGE	Two cameras on the head of the robot	RL	80%	Two Deep RL algorithms: Deep P-Network (DPN) and Dueling Deep P-Network (DDPN)
Matas et al. [63]	Aa combination of state-of-the-art of deep RL algorithms to solve the problem of manipulating deformable objects./The robot folds a towel up to a mark, folds a face towel diagonally, and drapes a piece of cloth over a hanger	Multiple tasks	7 DOF Kinova	Genius C170 web camera	RL	Diagonal folding 90% Hanging 77% Tape 86%	Deep deterministic policy gradient (DDPG) algorithm

**Fig. 2:**
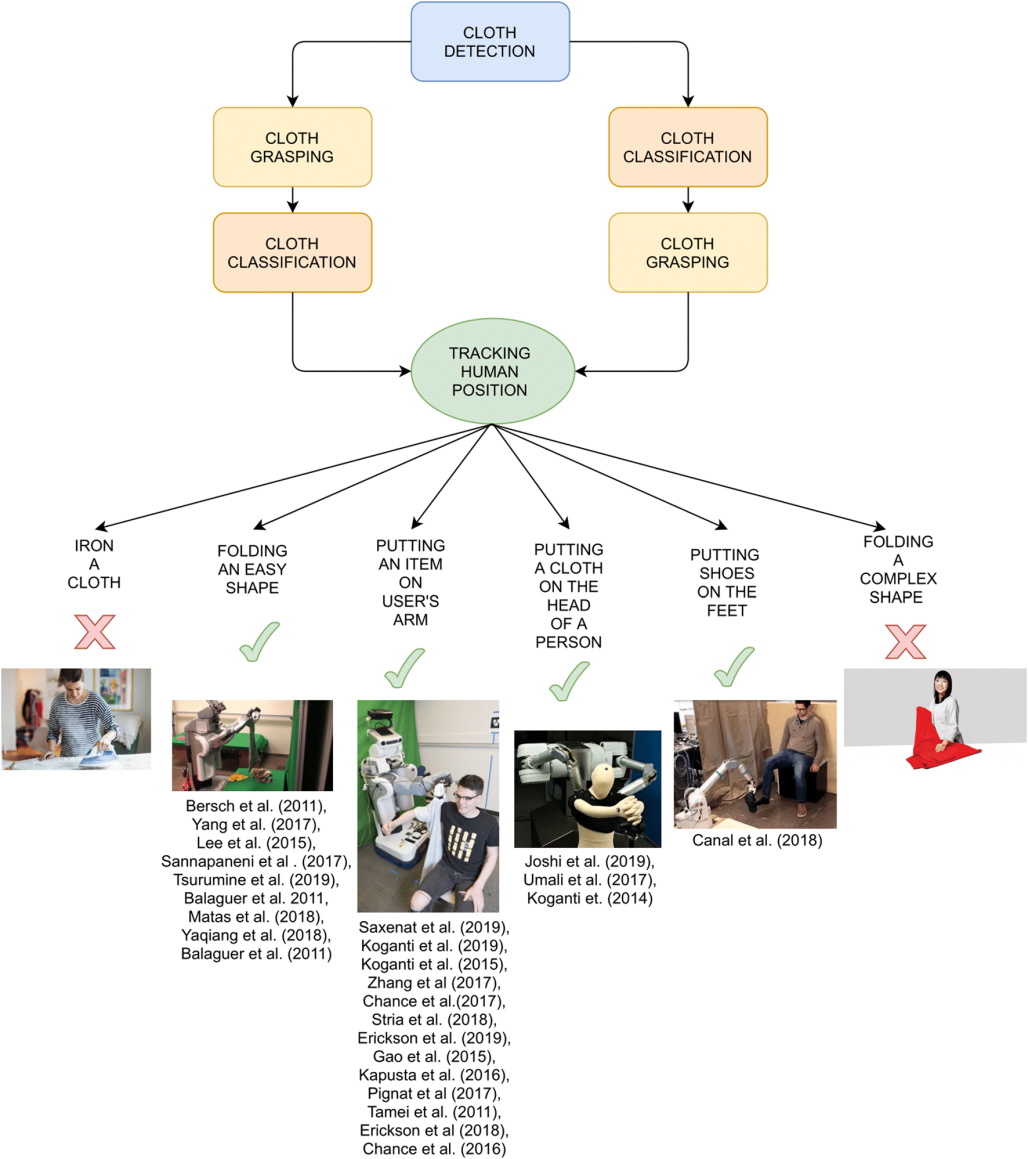
Robot assisted dressing process

In Fig. 2, the robot assisted dressing process is described. The first step is the cloth detection, followed by cloth classification and manipulation planning. The human position is then tracked to avoid the robot hurting the patient during the dressing task. Finally, different tasks accomplished by the robot are described in green, while the ones that should be investigated in the future are crossed in red. The tasks accomplished in several.

**Fig. 3:**
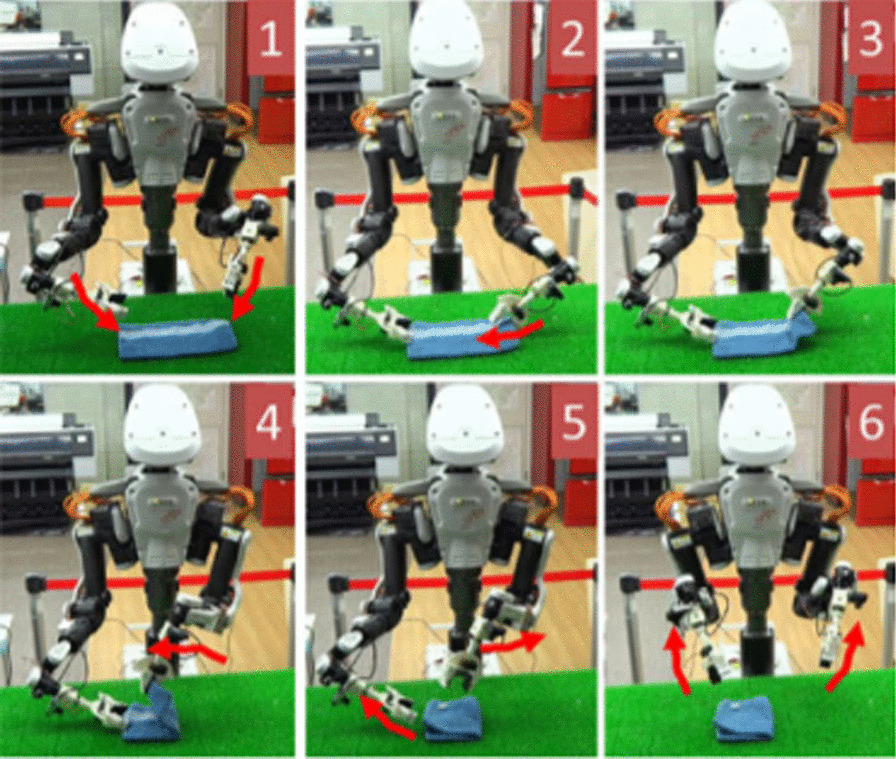
**a** Robot folding cloths using a SL strategy [23]

papers by robots are putting a t-shirt or a jacket on the arm of the user, putting a t-shirt on the head of a person, putting a shoe on the feet of the user, or dressing trousers. The tasks that should be accomplished in the future are, for example, folding a complex shape or ironing a garment.

### Cloth folding/untangling/coveraging

In this section, the papers concerning cloth folding, untangling or coveraging are evaluated and divided into subsections according to the control approach applied.

#### Supervised learning

In SL, each example is a pair consisting of an input object (typically a vector) and a desired output value (also called the supervisory signal). An SL algorithm analyses the training data and produces an inferred function, which can be used for mapping new examples [17]. Bersch et al. [24] used a DL approach with a PR2 robot for cloth manipulation and specifically for laundry folding. The quality of each grasp pose was evaluated using a function that calculates a score based on a set of geometric features and the score function was automatically trained using an SVM. Other strategies belonging to SL were the ones developed by Lui al. [25] that used a learning algorithm based on max-margin learning to manipulate deformable objects such as ropes with a PR2. Starting with a point-cloud obtained from an RGB-D camera, the authors designed appropriate features that their learning algorithm uses to first infer the rope’s knot structure and then chooses an appropriate manipulation action to untangle the knots in the ropes. Concerning humanoid robots, Yang et al. [23] also used DL to let a humanoid robot achieve folding task skills. The proposed approach was a real-time user interface with a monitor and provided a first-person perspective using a head-mounted display. Through this interface, teleoperation was used for collecting task operating data, especially for tasks that are difficult to be applied with a conventional method. A DL model was also utilized in the proposed approach. A deep convolutional autoencoder extracted image features and reconstructed images, and a fully connected deep time delay neural network learnt the dynamics of a robot task process from the extracted image features and motion angle signals (Fig. 3).

Tanaka et al. [14] used, a particular NN called Encode-Manipulate-Decode Network (EMD Net) for cloth folding. This EMD Net is essentially a 3D convolutional auto-encoder (providing the encoder and decoder modules), with a fully connected network (the manipulation module) inserted at the bottleneck layer.

Furthermore, in Hu et al. [26], limitation of movements of the user (modelled with the Gaussian Process Latent Variable Model) were studied and related to the online update of the dressing trajectory. The authors validated their idea by letting the robot fold towels.

A different approach was used by Jia et al. [27]. The authors used a random forest approach. They used imitation data consisting of visual features and control signals to learn a random forest controller that maps the observed visual features from an RGB-D camera to optimal control signals of a robotic manipulator to manipulate clothes. The controller parameters are learnt in two steps: online dataset sampling and controller optimization. The dataset is generated from an expert (a ground-truth hard coded control algorithm in their case but can also be a human) performing the manipulation task and RGB-D images from a camera collected which are then transformed into a low dimensional feature space by computing HOW features [27]. The random forest imitation learning controller parameters are learnt in an online fashion where a set of cloth simulation trajectories are first generated. During each time step of these trajectories, they query their expert for an optimal control action. This action is combined with the action proposed by the random forest controller and fed into the simulator to generate a new observation. This process is repeated until the imitation learning has converged to an optimal solution. The authors validated their approach by folding towels.

Finally, Corona et al. [28] used a hierarchy of three CNNs with different levels of specialization to grasp a garment and fold it using a Wam Robot. First, one robot arm grasps a garment from any point and shows it to an RGB-D camera and the cloth is recognized using the first CNN. Then, the visibility and locations of two reference grasping points are identified using the second CNN. Next, they located the second point of grasping with a third, more specialized, CNN.

#### Learning from demonstration

Learning from demonstration is conceived to provide and transfer assistive skills from non-expert users to the robot. It can be achieved using a kinaesthetic teaching or motion capture system, that demonstrations of the task executed in several situations can be used to adapt new situations rapidly. For these reasons, LfD is widely used for robotic manipulation tasks such as assistive dressing, towels and ropes folding.

Sannapaneni et al. [29] proposed an algorithm that folds cloth using amrita dual anthropomorphic manipulator (ADAM). Cloth coordinates (composed of four points) are extracted using depth images and are used to classify the cloth shape as a trouser and t-shirt. The main algorithm is to use marker coordinates along with cloth dimension and type stored. The marker is composed of four picks and place points, and then it is applied for folding the cloth by simple geometry calculation. It is implemented for cloth folding, but the limitation of this proposed LfD algorithm can only be used for a specific shape of robotic clothing assistance. To develop more complex assistive dressing algorithms, well-known LfD algorithms, which represent Dynamic Movement Primitives (DMP) and hidden Markov model (HMM) with a combination of the traditional methods, are applied.

#### Reinforcement learning

Balaguer et al. in [30] and in [31] were one of the first groups of researchers to formulate the cloth assistance problems as an RL problem. They combined imitation and RL to learn a control policy for two independent manipulators, working collaboratively, to achieve a towel folding task. Imitation training data was acquired by motion capture system detecting tracking the reflective markers placed on the towel and a human performing momentum folds—the kind of fold where the force applied to grasping points on the towel is used to give momentum to the towel and lay half of it flat on the table. Rewards were computed as the exponential function of the negative smallest error between an observation and training samples. This error was calculated by the Iterative Closest Point ICP (algorithm). PoWER [32], which was a state-of-art algorithm RL algorithm, was used to learn a policy based on the human samples.

Yaqiang et al. [33] also targeted to accomplish a t-shirt folding task while it is surmounted on the chest of a human being. They teach the general motion expected of a dual-arm robot by a teaching approach. A human demonstration of the expected folding behaviour is captured by a 3D range image capture system. Coloured markers placed on the shirt help recognize the state and shape of the cloth. Since the final state of the cloth is explicitly defined by the marker positions, the problem is reduced to a search problem and so the PILCO algorithm [34] is used for policy search.

Finally, Wu et al. [35] proposed a conditional learning approach for learning to fold deformable objects, improving sample complexity.

### Putting a cloth on user’s arm

In this part, the papers concerning wearing a cloth on the user's arm are evaluated and divided into subsections according to the control strategy applied.

#### Supervised learning

Zhang et al. [36] proposed the offline learning of a cloth dynamics model by incorporating reliable motion capture data and applied this model for the online tracking of human-cloth relationship using a depth sensor. The authors tested the approach using a robot that wears a cloth on the user's arm. Furthermore, Chance et al. [37] used the Support Vector Machine (SVM) to dress a jacket onto a mannequin or human participants, considering several combinations of user pose and clothing type. In detail, their SVM method involved searching for an optimal hyperplane that separates the data by class and is optimized by finding the largest margin at the boundaries.

Moreover, Stria et al. [38] used the SVM for the classification of garment categories and focuses particularly on putting a shirt on a user's arm.

Erickson et al. [39], used a fully connected NN that estimated the local pose of a human limb in real time. A key benefit of this sensing method is that it can sense the limb through opaque materials, including fabrics and wet cloth creating a robot that can assist a person during dressing and bathing tasks. The authors tested their approach by putting a hospital gown on the user's arm.

Finally, Gao et al. [40] used a random forest approach. They presented an end-to-end approach to build user specific models for home-environment humanoid robots to provide personalised dressing assistance (a robot puts a cloth on the user's arm). By mounting a depth camera on top of the head of a Baxter humanoid robot, they recognised the upper body pose of users from a single depth image using randomised decision forests. From sequences of upper-body movements, the movement space of each upper-body joint is modelled as a mixture of Gaussian learned by an expectation–maximization (EM) algorithm. The experimental results showed that their method of modelling upper-body joint movement of users, combined with real-time human upper body pose recognition enables a humanoid robot to provide personalised dressing assistance and has potential use in rehabilitation robotics and long-term human–robot interactions.

#### Learning from demonstration

Pignat et al. [41] proposed a different approach that encodes a joint distribution in a hidden semi-Markov model (HSMM) for adaptive dressing assistance. The parameters of this model, which represents the sequence of complex behaviours, were learned from human demonstration data using an EM algorithm. This method provided a solution for movement primitives (MPs), which are usually encoding only motor commands. Also, it increased the performance of robot behaviour that could be controlled both time-dependently and independently. Also, another HMM [42] method was used to classify the time series of forces robot-assisted dressing [43]. To classify the force, HMM was used for pattern recognition of the forces. Mainly raw forces were measured and the movement of the end-effector in the x and z direction was provided as the dataset from 12 human participants. The performance of the HMMs was validated using univariate and bivariate models with force in the x-direction. The limitation of these methods (DMP and HMM) was that the workspace where the robot can move for assisting dressing was inadequate compared to human body movement. In addition, demonstrations for a specific task like the one-to-one relationship had the restriction that motor commands were always linked to a unique perception distribution. To overcome this problem, the combination of each demonstration and point cloud scene was developed for folding towel manipulation. First, the method recorded the demonstrated pose and force trajectories. During the demonstration, the authors found that five demonstrations were sufficient for achieving generalization. Also, the point cloud of the scene was retrieved using a Kinect depth sensor at the beginning of each demonstration. The thin-plate spline robust point matching (TPS-RPM) algorithm [56] was used to match from each of the demonstrations to the current point cloud scene. After the demonstration, a mean trajectory and a sequence of time-varying feedback gains were extracted, and the gains were learned using a joint Gaussian distribution. This method is beneficial for dressing from small demonstrations, and point cloud scene well recognizes the new situation, but it still needs to obtain optimal gains to optimize the task.

Kapusta et al. [44] provided evidence for the value of data-driven haptic perception for robot-assisted dressing through a carefully controlled experiment. To design an informative and replicable experiment, they deliberately focused on a representative sub-task of dressing with a commonly used article of clothing, and they tested their approach by wearing a hospital gown on the user's arm.

#### Reinforcement learning

Clegg et al. [45] approached the dressing problem differently by viewing the long horizon task of dressing as a sequence of smaller sub tasks. They have argued that learning to dress is challenging because humans rely heavily on haptic information and the task itself is a prolonged sequence of motions which are very costly to learn together especially in the right order. They have thus proposed to learn a unique policy for each subtask and have introduced a policy sequencing algorithm that matches the output state distribution of one subtask to the input state distribution of the other subtask while the transitions between the different subtasks are managed by a state machine. To deal with a high dimensional observation space typically associated with dressing tasks, they defined their observation space as a 163-dimensional vector which includes information on the human’s joint angles, garment feature (e.g. a sleeve opening) locations, haptics (contact forces between human and cloth), surface information (information on the inner and outer surfaces of the garment) and a task vector. The reward function is then defined as the weighted sum of the progress reward (extent to which a limb is dressed), deformation penalty (penalization of undesired cloth deformation), geodesic reward, reward for moving the end effector in the direction of the task vector and another reward that attracts the character to a target position. With these definitions of the reward and state which are queried from a dressing simulation, Trust Region Policy Optimization algorithm (TRPO) [46] was used to update the policy parameters represented by a neural network. They validated their approach by putting a hospital gown on a virtual user. The same authors presented a DRL based approach for modelling collaborative strategies for robot-assisted dressing tasks in simulation. Their approach applied co-optimization to enable distinct robot and human policies to explore the space of joint solutions to maximize a shared reward. In addition, they presented a strategy for modelling impairments in human capability. They demonstrated that their approach enables a robot, unaware of the exact capability of the human, to assist with dressing tasks.

#### Other methods

Chance et al. [47] created strategies for an assistive robot to support dressing using a compliant robotic arm on a mannequin. A tracking system is used to find the arm position of the mannequin and it supports trajectory planning using waypoints. Torque feedback and sensor tag data provide failure detection. Also, speech commands are allowed for correction of detected dressing errors successfully. The authors tested on ten different poses of the mannequin with the proposed method, and it showed that assistive dressing tasks could be developed without complex learning algorithms. Further, the method investigated has the advantage of using a small number of low-cost sensors which can be used to sense unplanned movement in smooth trajectories. The problem of this strategy was to not have force feedback from the mannequin that is important to know (people could be hurt by the robot). They validated their approach by putting a t-shirt and a jumper on the user's arm.

Erickson et al. [48] showed how task-specific LSTMs can estimate force magnitudes along a human limb for two simulated dressing tasks. At each time step their LSTM networks took a 9-dimensional input vector consisting of the force and torque applied to the end effector by the garment and the velocity of the end effector. The networks then output a force map at each time step consisting of hundreds of inferred force magnitudes across the person’s body. Their work was tested on a simulated robot that puts a shirt on a virtual user’s arm.

### Putting a cloth on user’s head

In this section, the papers concerning wearing a cloth on the user's head are evaluated and divided into subsections according to the control strategy applied.

#### Supervised learning

Koganti et al. [49] proposed a data-efficient representation to encode task-specific motor-skills of the robot using Bayesian non-parametric latent variable models to learn a dynamics model of the human-cloth relationship and use this model as a prior for robust tracking in real-time. They reduced their policy search space by first learning a low dimensional latent space using the BGPLVM [44]. A dataset of successful clothing assistance trajectories was then used to train a latent space that encodes the motor skills. Each of the trajectories were then transformed into a sequence of points in the latent space forming latent space trajectories followed by searching for policy using the PoWER algorithm [32]. The authors validated their idea by wearing a t-shirt on a person. The same authors learnt the underlying cloth dynamics using the shared Gaussian Process Latent Variable Model and by incorporating accurate state information obtained from the motion capture system into the dynamics model. Shared GP-LVM provides a probabilistic framework to infer the accurate cloth state from the noisy depth sensor readings. The experimental results showed that shared GP-LVM was able to learn reliable motion models of the T-shirt state for robotic clothing assistance tasks. They also demonstrated three key factors that contribute to the performance of the trained dynamics model. The advantage of using GP-LVM is that a corresponding latent space manifold can be learned for any representation used in the observation spaces.

Saxena et al. [50] also used SL for grasping point detection and for garment recognition; the challenge of their work was to use the Kinect camera near the garment to try the algorithm with an occluded vision of the object. The authors tested their approach by putting a t-shirt on a person.

#### Learning from demonstration

**Fig. 4:**
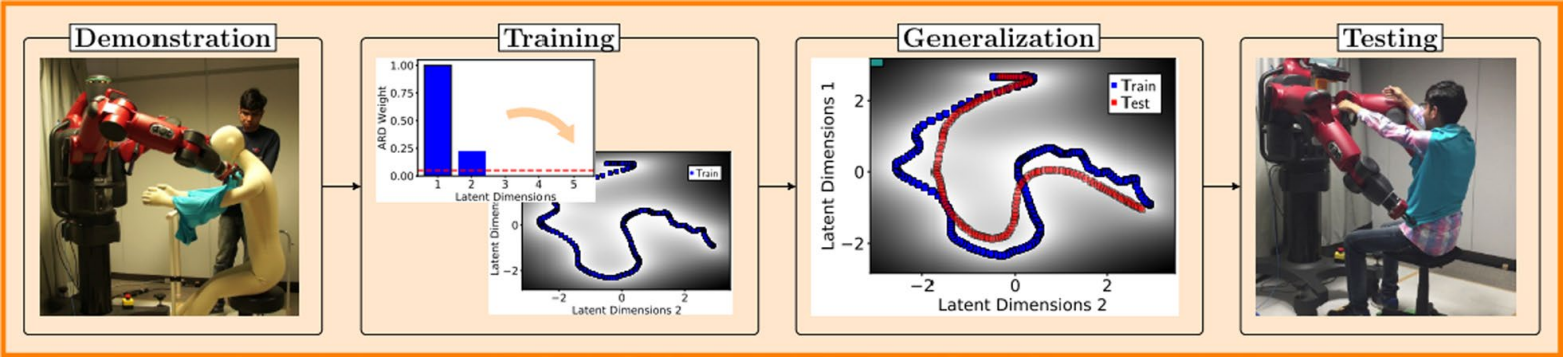
LfD example where a Baxter robot is dressing a man with a T-shirt [51]

Joshi et al. [51] (Fig. 4) presented a framework for robotic clothing assistance by DMP on a Baxter robot. The authors divided the dressing task into three phases (reaching, arm dressing, and body dressing) and each phase was applied for different skills. The reaching phase was to move the robot arm in a specific location without collision, thus it can be achieved through point-to-point trajectory while the arm dressing phase was to reach the ends at the elbow position. To make the robotic arm reaching the position DMP, which can be applied for a global trajectory modification, was used. DMP parameters can be acquired from the kinaesthetic demonstration, and support generating a trajectory globally using the start and goal parameters of DMP. Compared to reaching the elbow position, generating a trajectory to the torso position is more complicated, thus the authors introduced the Bayesian Gaussian Process Latent Variable Model (BGPLVM) as the body dressing phase. They applied BGPLVM to encode complicated motor-skills to generalize trajectory in latent space and modify the trajectory locally. The authors validated their idea using a manipulator that puts a t-shirt on a person.

#### Reinforcement learning

Koganti et al. [52] used a depth sensor to extract and filter a point cloud of the t-shirt collar and sleeve which in turn were detected by a colour extraction method. Once retrieved, both the point clouds were approximated with an ellipse followed by computing the same topological relationship but this time, in real-time. They also modified the reward function to now calculate the Mahalanobois distance between the current and the target states to account for the different scales of different state variables. The authors tested their model using a robot that puts a t-shirt on the head of the person. Twardon et al. [53], instead, made a dual-arm robot, attached with anthropomorphic hands, and learned to put a knit cap on a styrofoam head. They modelled the head as an ellipsoid using point cloud data and constructed a head-centric policy space where the policy search takes place. The policy was then defined in this space as the parameterized end-effector trajectories (parameterized as B-splines) from the back of the head (back pole) to its front (front pole). They then defined an objective function which gives the robot a fixed reward for successful task completion while supporting the robot to find a trade-off between minimizing the risk of early failure and establishing contact between the fabric and the head. All this setting allowed the authors to use a gradient-free direct policy search approach to find the optimal policy by minimizing the objective function Active-CMA-ES algorithm [80].

Furthermore, Tamei et al. [54] presented a novel learning system for an anthropomorphic dual-arm robot to perform the clothing assistance task. The keys of their system were to apply a reinforcement learning method for coping with the posture variation of the assisted person, and to define a low-dimensional state representation utilizing the topological relationship between the assisted person and the non-rigid material. With their developed experimental system for T-shirt clothing assistance, including an anthropomorphic dual-arm robot and a soft mannequin, they demonstrated the robot quickly learns to modify its arm motion to put the mannequin’s head into a T-shirt.

Additionally, Matsubara et al. [55] and Shinoara et al. [56] proposed a novel learning framework for learning motor skills for interacting with non-rigid materials by RL. Their learning framework focuses on the topological relationship between the configuration of the robot and the non-rigid material. They constructed an experimental setting with an anthropomorphic dual-arm robot and a tailor-made T-shirt for the robot. They both applied the method to the robot to perform the motor task of wearing a T-shirt.

#### Other methods

Klee et al. [57] focused on the motion interaction between the robot and the person. The authors found a solution involving manipulator motions and user repositioning requests. Specifically, the solution allows the robot and user to take turns moving in the same space and is cognizant of the user’s limitations. To accomplish this, a vision module monitors the human’s motion, determines if they are following the repositioning requests, and infers mobility limitations when they cannot. The learned constraints were used during future dressing episodes to personalize the repositioning requests. Their contributions included a turn-taking approach to human–robot coordination for the dressing problem and a vision module capable of learning user limitations. They validated their approach using a robot that puts a hat on the user’s head.

### Putting a shoe on user feet

In this section, the papers concerning wearing a shoe on the user's feet are evaluated and divided into subsections according to the control strategy applied.

#### Learning from demonstration

Canal et al. [58] defined a method to guide a planner to choose the preferred actions by the user. The user model was included in the planning domain as predicates, and the actions’ associated costs depend on them, the costliest actions being those that do not satisfy the user model. Moreover, they used a stochastic planner with NID rules that contemplate the possibility of different action outcomes and failures. The initial user model was inferred by asking two simple questions to the user, related to his/her confidence and comfortability. A Fuzzy Inference System (FIS) was then used to translate the answers to planning predicates. To make the planner adapt to user behaviour change and to cope with wrongly inferred user models, each rule’s probabilities and costs were updated. First, an initial refinement was performed to favour the inferred user model. Then, after each task completion, the satisfaction of the user was used to refine each rule cost, and the outcome of each action was used to refine the success’ probabilities. This defines a separation between the user model and the action outcomes, as the user delight should not be measured only by the success of the actions, which may fail due to events unrelated to the users’ preferences. Moreover, the system was able to plan with task related actions as well as with interaction actions, asking the user to move when needed and informing them regarding the next action when this increased the success rate of the action. They showed how the system was able to adapt to user behaviour changes, as well as how the use of feedback to update the action costs with the decreasing m-estimate produced a more stable behaviour and faster convergence to the preferred solution.

### Putting an item on user leg

In this part, the papers concerning wearing a cloth on the user's leg are evaluated and divided into subsections according to the control strategy applied.

#### Other method

Yamazaki et al. [59] focused on a different task: the actions by which the robot can pull a pair of trousers along the subject’s legs. These actions are frequently demanded by humans requiring dressing assistance and which are potentially automatable. To overcome this problem the authors implemented the dressing procedure using a life-sized humanoid robot. Estimating the shape of the legs from images captured by a three-dimensional range camera, they proposed a method of modifying the trajectory from the basic one estimated from statistical human-body data.

### Multiple tasks

In this section, the papers multiple tasks are evaluated and divided into subsections according to the control strategy applied.

#### Learning from demonstration

Lee et al. [5] presented an approach for generalizing force-based demonstrations of deformable object manipulation skills to novel situations. Their method uses non-linear geometric warping based on point cloud registration to adapt the demonstrations to a novel test scene, and then learns appropriate feedback gains to trade off position and force goals in a manner consistent with the data, providing for variable impedance control. Their results showed that including forces in the manipulation tasks allows for significantly greater generalization than purely kinematic execution: knots could be tightened more tightly in ropes with greater length variation and could be tied to a pipe without slipping off, towels of varying geometries could be stretched and laid flat, and whiteboards could be erased effectively. They chose their tasks to include both phases that were determined primarily by pose, such as positioning the gripper to grasp the rope, and phases that were primarily force-driven, such as tightening the knot. Performing such tasks kinematically is unreliable, because some parts are defined primarily by the force exerted on the object, while others require precise positioning. Automatically determining whether force or pose is important at each phase is essential for effectively generalizing demonstrations of such tasks. The authors validated their work using a robot that tied a knot, folded a towel, erased a whiteboard, and tied a rope to a pipe.

#### Reinforcement learning

**Fig. 5:**
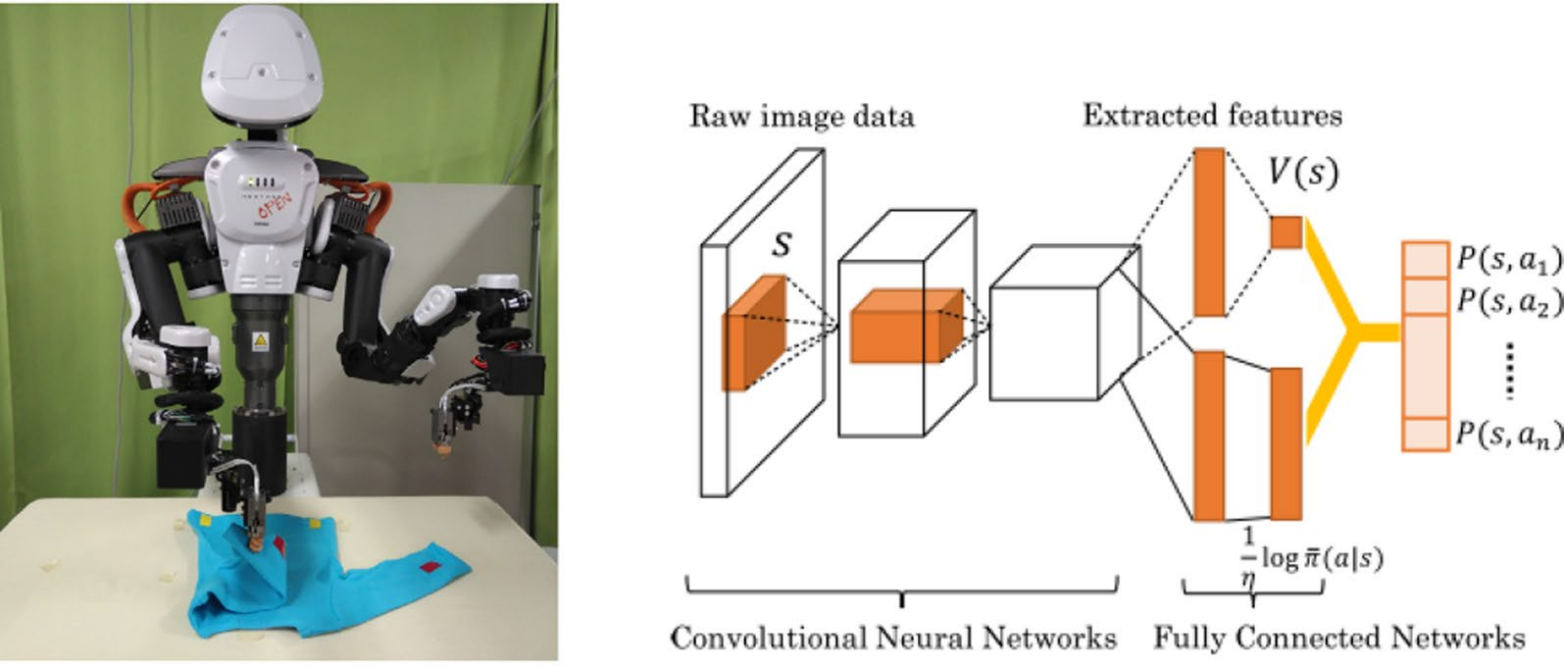
**a** RL example of a robot that is folding a T-shirt [57] and (**b**) RL example of network implementation with folding steps of the T-shirt [57]

Tsurumine et al. [60] (see Fig. 5a, b) proposed two DRL algorithms: deep policy network and duelling deep policy network structure which combine the nature of smooth policy update with the capability of automatic feature extraction in deep neural networks to enhance the sample efficiency and learning stability with fewer samples. To exploit the nature of smooth policy update, they used dynamic policy programming [61] which considers the Kullback–Leibler divergence between current policy π and baseline policy π̄ into the reward function to minimize the difference between the current and baseline policy while maximizing the expected reward. A DDQN inspired novel architecture was also presented that learned separate value and advantage functions and then used human demonstrations to drastically reduce the exploration space for their RL agent. Their state was defined as raw RGB images which are then mapped to optimal actions by the neural network. Results reported, indicated a stable and sample efficient learning for cloth manipulation tasks such as folding a t-shirt and flipping a handkerchief when compared to deep Q-learning (DQN) [62] algorithm while simultaneously earning higher total reward. The robot in this approach tied a knot, folded a towel, erased a whiteboard, and tied a rope to a pipe.

Matas et al. [63] instead, proposed a task agnostic algorithm based on Deep RL which bypasses the need to explicitly model cloth behaviour and does not require reward shaping to converge. The agent was able to learn 3 long horizon tasks: folding a towel to a tape mark, diagonal folding of face towel and draping a small towel over a hanger. Training was seeded with 20 demonstrations and happened entirely in simulation with a couple of adaptations to account for imperfections in experimental deformable body support, and with domain randomization to enable easy transfer of the policy. The learning algorithm incorporated 9 improvements proposed in the recent literature and they presented ablation studies to understand the role of these improvements. The robot in this approach folded up a towel up to a mark, folded a face towel diagonally, and draped a piece of cloth over a hanger.

## Discussion

As the description of the result section, the three learning-based control approaches have lots of advantages for cloth manipulation and dressing assistance. However, it still has disadvantages that needs to improve the approaches, so the pros and cons of these approaches are described in Table 2. We also analyse the state of the art that provides a list of hints regarding control approaches during dressing tasks. Future research efforts should lead to overcoming the limitations of the existing works, as summarized in Table 3. It identifies several areas that should be analysed in future works such as dataset, multisensory approach, perception, manipulation, simulation, and experimental phases. Moreover, legal, and social aspects should be taken into consideration to build an efficient behavioural model for future robots.

**Table 2:** Pros and cons of SL, LfD, and RL

	Pros	Cons
SL	1. This approach is very accurate for seen clothes recognition using the training dataset [50] 2. Given data and labels is very helpful for classification and detecting clothes grasping points for the cloth application [64]	1. Without prior knowledge about specific garments, clothes classification and recognition is inaccurate [64] 2. It is not easy to extend to complex cloth manipulation scenarios [65]
LfD	1. This approach can transfer the learned motion to unseen scenarios [4] 2. The demonstration provides a high-level plan that is used to execute low-level control for cloth manipulation [66]	1. This approach mostly focuses on a single task, which means it is difficult to learn to manipulate new objects [66] 2. After the demonstration, this approach is not easy to adapt different scales or shape of robotic clothing assistants.[67] [29]
RL	1. This approach makes it possible to accomplish complex clothing assistance tasks using appropriate reward functions [60] 2. It is the only way to collect data for interacting with the environment [68]	1. It is still challenging to find suitable reward functions for complex cloth manipulation without reward shaping [60] 2. This approach requires a lot of data which makes the computation time consuming which, in turn, increases the difficulty to achieve the results [68]

**Table 3:** Challenges and opportunity/Weaknesses

Type	Keywords of barriers/limitations	Challenges and opportunities/weaknesses
Dataset	Dataset limitation [52, 56, 65]	Large datasets with different categories of clothes should be created to avoid the dataset limitation. More information is given to networks, the easier is for them to produce good results
Sensor technology	Deep studies on the integration of sensor technology [20, 33, 56]	A multisensory approach should be used to acquire as much as information the robot needs to accomplish its task in real-time (not only use vision but also using force and tactile inputs)
Perception	Relying only on vision for the detection of clothes [20]	One possibility not to relay only vision is to take an active perception approach, e.g., turn the cloth over or introduce additional slack for perceiving it better. Moreover, tactile sensing could also help in better perception. This kind of approach is used only in few papers as [69] and should be developed more to achieve better results
	No combination between optical flow with forces and 3D information [55]	Feature description combining optical flow with force data should be studied because significant relationships between force data and optical-flow data could improve the success rate and accuracy of failure detection and recovery
Manipulation	Robots could learn to infer forces exerted on humans	Robots could learn to infer the forces that people physically feel during robot- assisted dressing to have a more real dressing scenario
	Occlusion of the cloth [52]	To solve the issue of the occlusion of cloth, we could add additional views of it using a hand-mounted camera or putting more cameras around the grasping scenario
	In many works the authors concentrate only on a single scenario [34, 40]	Systems should not only perform upper or lower body tasks but should do both and their integration could bring balance into the controller. Moreover, our framework could enable robotic assistance in other dressing tasks such as undressing the person
	The object grasped is unknow by the robot [30]	In principle, the limitation of not knowing the object grasped could be overcome simply by collecting data from many object manipulation scenarios, so as to learn a single model that generalizes effectively across objects. A more nuanced approach might involve correlating the behaviour of objects in the human demonstrations with other previously experienced manipulations, to put them into correspondence and infer the behaviour of an object for which prior experience is unavailable
Experimental and simulation phase	A limitation of some studies is that a soft mannequin is used as a subject or simulating only the dressing task [49, 56, 70, 71]	The robot should work with a real person so that researchers can have feedback from them about the force applied by the robot or other problems that can happen during the dressing task to overcome the limitation of using the robot only in simulation
	Neural Network limitations [65]	Comparing neural networks to see the difference between them and find the better and fast approach to accomplish the task
	Better planning algorithms [25]	Algorithms that consider the limitations of the arms movements of a robot as well as uncertainty in perception would also improve the performance and the safety of the people that are working with a robot
	Improves manipulators trajectories [57, 72]	Improving manipulator trajectories should be studied in the future to make the robot more user friendly and to reduce the computation time
	Autonomy of the robot [57]	The robot should be as autonomous as possible to reduce the computation time. For example, when a robot finds a goal infeasible, it should not request the user to reposition his or herself but should recompute autonomously its trajectory
	Lack of support for deformable objects in most robotic simulators [28]	Create accurate models of deformable object grasping, incorporate it into widely used simulators and release the environments to create a set of benchmark tasks for future research in the domain
	Use of markers attached to clothes [30, 33]	Rewards calculated based on markers attached to the clothes which is not a real-world scenario
	Single scenario [44, 58]	Having different scenarios could improve the quality of the robot because it could adapt it to several situations instead of having only a single scenario
Legal and safety aspects	No rules can be found in the legal or safety fields of social robotics [50]	The birth of a regulation for social robots could be an important step to overcome the problem that social robots can’t operate in environments with people without a supervision of an operator Moreover, the safety of user should be the foremost priority

On the other hand, it should be noted that research and development in this field is following a positive trend with many examples of concrete experimentation with code availability for users, which can be found [30, 44, 56]. This is very useful for the reader if they want to replicate and develop new code.

### Dataset

A crucial aspect in dressing a person using a robot or more technically speaking, using machine learning techniques, is the acquisition of the dataset of clothes and this situation brings to a limitation of the dataset itself. In the state of the art, we can find several apparel datasets such as the DeepFashion dataset or Fashion-MNIST dataset; the issue is that those datasets are very small compared to other existing datasets not concerning fashion, such as the MNIST dataset. Furthermore, in [51], the authors discussed that their approach (dressing a sleeveless t-shirt) should be extended to other clothing articles such as pants, jackets and so on, for each type of clothing article. The same arguments were discussed in [28] where the authors explained that in their future work, they would design a system that can be easily scaled to work with more types of garments with few modifications to expand their dataset. The main problem of having a small dataset is that this brings to a wrong classification of clothes when a new item is used in the experimental phase because the robot is not able to recognize it.

### Sensor technology

Sensor technology plays an important role in the interaction between humans and the robot and as concerns fashion, many sensors can be found and several of them are being employed to infer the cloth state in cloth manipulation and assisted dressing tasks. In the field analysed, sensor information is often used separately while it is important to have a multisensory approach to improve the accuracy of the system. Zhang et al.[36], for example, would aim in their future work to combine multi-modal information, including gripper positions, force information and depth images, into a probabilistic framework to obtain real-time estimate of the arm pose during the dressing process.

### Perception

A second ability that should be deeply analysed is the area of perception. At present, perception is mostly relying on vision for the detection of clothes, and this is a very limited view for a robot. There are few works such as Yuan et al.[73], where information from tactile sensors is used for perception. Stria et al. [38] underlined the importance of not relying only on vision in the field of perception and they stated that they have future plans to develop advanced models of clothing taking into account their physical properties when unfolding a towel. They also pointed out that they plan to detect and model special parts of clothing like buttons, pockets or collars which provide additional information about the garment configuration.

Furthermore, in [25], the garment taken by the robot is put in a specific position which represents a very limited scenario. The authors stated that active perception is needed for identifying rope’s knot structure, e.g., turning the rope over or introducing additional slack for perceiving it better. Moreover, they pointed out that tactile sensing could also improve perception. Saxena et al. [50] proposed to add additional views of the cloth using a hand-mounted camera or putting more cameras around the grasping scenario to avoid occlusion and to have an improved representation of the item.

### Manipulation

There are many issues to be solved concerning manipulation. The main problem that can be found during dressing tasks is clothing assistance that is developed only for specific scenarios.

Another important aspect is the number of experiments and of people involved in the trials.

Tamei et al. [54] also underlined the importance of having a validation of putting in a mannequin’s head a t-shirt with more participants. They stated that testing their method of dressing with different participants is important to obtain more information about the experience of the person regarding the interaction between him/her and the robot tested to improve it.

Furthermore, another issue that should be considered, is to manipulate not only one object at a time, but instead collecting more clothes at the same time even if some parts of them are occluded [28]. Collecting more apparels, would speed up the process of the dressing assistance.

Manipulation in situations of occlusion, is another problem that should be solved as stated in and in [36].

Another important issue is that some methods cannot learn to manipulate new objects exclusively by watching human demonstrations, since performing a manipulation task requires a model that can effectively predict the motion of the object, and this model is learned from the robot’s own experience. Yang et al. [23] underlined this concept stating that their future focus is to implement their model on unknown items and in a quick manner. This limitation could be overcome simply by collecting data from many object manipulation scenarios.

### Simulation and experimental phases

In the simulation phase, an issue that should be overcome is the lack of support for deformable objects that most robotic simulators have. In literature there are some simulators like Gazebo, among the widely used simulators, [74] cannot offer this specific, only Pybullet [75] implements some rudimentary and experimental functionality for simulating deformable objects. Solving this problem would let the researchers create accurate models of deformable objects grasping that can be useful to succeed in the experimental phase. One problem is that in many real scenarios, the model of the robot is tested on a mannequin. Working with it represents a problem, since the researchers cannot have feedback from the mannequin about the intensity of the forces applied by the robot [54]. If the robot, instead, interacts with a person, it can receive all this kind of feedback. A second issue is related to the importance of having several scenarios for validating the behaviour of a robot [44]. During the experiments of this paper a specific scenario was an armrest supported the participant’s upper arm, and the participant’s forearm was initially aligned to the robot’s motion. In other dressing tasks, body parts could have greater freedom to move with respect to the robot, resulting in more variability of the forces measured by the robot. Likewise, a robot might hold a garment in place while the person’s body moves, which might increase the variability of the forces measured by the robot [44]. Yang et al. [23] also underlined the importance of having several scenarios stating that they expected that the accuracy and adaptability for various environments and the robot’s task performing speed should improve in the future.

Another important issue that emerges in [57], is the importance of the autonomy of the robot in terms of being controlled by humans. If a system is autonomous, the computation of time during tasks is reduced and the robot could achieve a better performance in dressing tasks. Moreover, in two papers the importance of the number [54] and feedback of participants is pointed out to increase the accuracy of the model. Other elements that should be considered during the experimental phase are the following: trying not only a single network but using several of them, comparing them and finding the one that boots the accuracy of the dressing task and having improved planning algorithm [20]. In the last case, algorithms that consider the limitations of the arms of a robot as well as uncertainty in perception would also improve the performance and the safety of the people who are working with a robot. Several works consider only a single dressing task, or a single object grasped, or specific configurations [55], and these limitations should be overcome in the future to have as many generic models as possible. Moreover, applying for such tasks as turning socks inside out, and applying bandages, [56] could be a new step in this field. Another issue is the conversion from mesh to voxel representations. For example, given a cloth folded neatly in half, it can be nearly impossible to distinguish on which side the fold is by looking at the voxel representation alone. They found that such ambiguity can be greatly reduced by adopting a coloured voxel representation that marks the cloth’s hems. However, in practical application this would require visual recognition of the hems in a pre-processing step. Canal et al. [58] stated the importance of long-term adaptation of the system. The authors explained that in the future long-term adaptation should be analysed carefully, as well as the inclusion of more actions and preferences, with the possibility of automatically learning the actions along with the preferences.*4.6. Legal aspect and safety aspects.*

At present, few rules can be found in the legal or safety fields of social robotics. In [76], expert opinions are given from different international workshops exploring ethical, legal, and social (ELS) issues associated with social robots, but many questions remain open. Several extensions to cope with the safety of patients must be made in several works such as [55] where it is pointed out that this topic should be analysed in depth. Finally, the birth of a regulation for social robots, like the one created for drones during the past years, could be an important step to have the possibility to use robots in crowded environments [77].

## Conclusions

This paper focuses on the control approaches that service robots use for dressing tasks. The current state-of-the-art of existing systems used in this field is presented to identify the pros and cons of each work with the aim to provide recommendations for future improvements.

Several issues must be solved to improve the development of robots for clothing assistance. First, there should be an increment of the size of the dataset to have a better training and to obtain better results. Concerning perception, most experiments focus on one or two specific skills and have been executed in pre-defined laboratory conditions. This is far from the human ability to approach and grasp items where needed, reverse inside-out sleeves or fold clothes [78]. Moreover, there is the need of high-resolution depth to solve to go towards more accurate wrinkle measuring and state estimation, but processing times are still too long for real-time applications. Furthermore, future solutions should include active vision, with the help of a robot moving base, to obtain multiple views of the garment and generate a more robust prediction [79]. Another aspect that should be taken into consideration in the future is using a multisensory approach to acquire as much as information the robot needs to accomplish its task in a fast way (not only use vision but also using force and tactile inputs).

Perceptual skills must gain in speed and accuracy and must be tightly coupled with manipulation to achieve active vision strategies to resolve uncertainties in an agile way.

Moreover, robots should have a multitasking strategy to not only accomplish a single task but to be more useful in different tasks (dressing both lower and upper limbs). The researchers should improve networks limitations, the autonomy of the robot, improve manipulators trajectory, the lack of support for deformable objects in most of the robotic simulators, and should test the robot in different scenarios with different populations, to receive feedbacks by the participants of an experiment. Manipulation of clothes should evolve to have algorithms that recognize unknown objects or occluded objects, and more attention should be paid to the forces applied from robots to a patient.

Furthermore, legal or safety fields for social robotics should be studied in deep since it is still a new topic of research.

Additionally, the tasks of state estimation and tracking require further advances in versatility and uncertainty handling to effectively mimicking human comprehension of cloth states and our intuitive discretization of what is a continuum of deformed states [78].

To maintain the importance of each contribution, it is fundamental to include in a whole vision all the suggestions provided by each work.

Finally, for what concerns the future direction of learning control strategies for dressing task it can be shown that Transformers [80, 81] could be used in the future for clothes classification tasks. Other models that could be applied in future works of RL, in the manipulation of clothes and dressing assistance, could be the one used in [82]. Then, for what concerns LfD in apparel manipulation and assistance, [83] and [84] could be used in future works.

Although many improvements remain to be accomplished, the already satisfying results the authors have achieved are an optimum starting point to develop a better solution using knowledge of human cognitive and psychological structures.

## Data Availability

Not applicable.

## Abbreviations

HRI: Human robot interaction
SL: Supervised learning
LfD: Learning from demonstration
RL: Reinforcement learning
DL: Deep learning
CNN: Convolutional neural network
NN: Neural network
ANN: Artificial neural network
LTSM: Network-long short-term memory
(EM*D): Encode-manipulate-decode
SVM: Support vector machines
IMU: Inertial measurement unit
UEM: Unsupervised expectation minimization
GMMs: Gaussian mixture models
GP-LVM: Gaussian process latent variable model
HMMs: Hidden Markov models
DMP: Dynamics movements primitives
HSMM: Hidden semi Markov models
MDP: Markov decision process
DPN: Deep-P-network
DDPN: Deep duelling-P-network
DDPG: Deep deterministic policy gradient
TRPO: Trust region policy optimization
MVP: Maximal value under placing
TPS-RPM: Thin-plate spline robust point matching
DDPGfD: Deep deterministic policy gradient from demonstration
DDQN: Double deep Q learning

## References

[1] Conti D, Trubia G, Buono S, Di Nuovo S, Di Nuovo, A. Evaluation of a robot-assisted therapy for children with autism and intellectual disability. In Proceedings of the Lecture Notes in Computer Science (including subseries Lecture Notes in Artificial Intelligence and Lecture Notes in Bioinformatics); 2018.

[2] Cavallo F, Semeraro F, Fiorini L, Magyar G, Sinčák P, Dario P (2018). Emotion modelling for social robotics applications: a review. J Bionic Eng.

[3] Sancarlo D, D’Onofrio G, Oscar J, Ricciardi F, Casey D, Murphy K, Giuliani F, Greco A. MARIO project: A multicenter survey about companion robot acceptability in caregivers of patients with dementia. In: Proceedings of the Lecture Notes in Electrical Engineering; 2017.

[4] Loi SM, Bennett A, Pearce M, Nguyen K, Lautenschlager NT, Khosla R, Velakoulis D (2018). A pilot study exploring staff acceptability of a socially assistive robot in a residential care facility that accommodates people under 65 years old. Int Psychogeriatrics.

[5] Lee AX, Lu H, Gupta A, Levine S, Abbeel P (2015). Learning force-based manipulation of deformable objects from multiple demonstrations. Proc IEEE Int Conf Robot Autom..

[6] Löffler D, Dörrenbächer J, Welge J, Hassenzahl M. Hybridity as design strategy for service robots to become domestic products. In: Proceedings of the Conference on Human Factors in Computing Systems Proceedings; 2020.

[7] Iglesias A, Jose RVA, Perez-Lorenzo M, Ting KLH, Tudela A, Marfil R, Duenas A, Bandera JP. Towards long term acceptance of Socially Assistive Robots in retirement houses: Use case definition. In: Proceedings of the 2020 IEEE International Conference on Autonomous Robot Systems and Competitions, ICARSC 2020; 2020.

[8] Limosani R, Manzi A, Fiorini L, Cavallo F, Dario P (2016). Enabling global robot navigation based on a cloud robotics approach. Int J Soc Robot.

[9] Gerłowska J, Skrobas U, Grabowska-Aleksandrowicz K, Korchut A, Szklener S, Szczȩśniak-Stańczyk D, Tzovaras D, Rejdak K (2018). Assessment of perceived attractiveness, usability, and societal impact of a multimodal Robotic Assistant for aging patients with memory impairments. Front Neurol.

[10] Cesta A, Cortellessa G, Orlandini A, Tiberio L (2016). Long-term evaluation of a telepresence robot for the elderly: methodology and ecological case study. Int J Soc Robot.

[11] Fasola, J, Mataric, M. A Socially Assistive Robot Exercise Coach for the Elderly. J Human–Robot Interact 2013.

[12] Fiorini L, Esposito R, Bonaccorsi M, Petrazzuolo C, Saponara F, Giannantonio R, De Petris G, Dario P, Cavallo F (2017). Enabling personalised medical support for chronic disease management through a hybrid robot-cloud approach. Auton Robots.

[13] Turchetti G, Micera S, Cavallo F, Odetti L, Dario P. Technology and innovative services. IEEE Pulse. 2011; 2. 10.1109/MPUL.2011.940428 21550871

[14] Vos AE (2009). Falling fertility rates: new challenges to the European welfare state. Socio-Economic Rev.

[15] Mcwilliam RA, Young HJ, Harville K (1996). Therapy services in early intervention: current status, barriers, and recommendations. Topics Early Child Spec Educ..

[16] Jiménez P, Torras C (2020). Perception of cloth in assistive robotic manipulation tasks. Nat Comput.

[17] Norvig SJR. Artificial intelligence a modern approach. 2nd Edn; 1996; ISBN 9780121619640.

[18] Kotsiantis SB (2007). Supervised machine learning: a review of classification techniques. Inform.

[19] Argall BD, Chernova S, Veloso M, Browning B (2009). A survey of robot learning from demonstration. Rob Auton Syst..

[20] Billard A, Siegwart R. Robot learning from demonstration. In: Proceedings of the Robotics and Autonomous Systems; 2004.

[21] Kaelbling LP, Littman ML, Moore AW (1996). Reinforcement learning: a survey. J Artif. Intell. Res..

[22] Levine S. Reinforcement learning and control as probabilistic inference: Tutorial and review. arXiv 2018.

[23] Yang PC, Sasaki K, Suzuki K, Kase K, Sugano S, Ogata T (2017). Repeatable folding task by humanoid robot worker using deep learning. IEEE Robot Autom Lett.

[24] Bersch, C, Pitzer, B, Kammel, S. Bimanual robotic cloth manipulation for laundry folding. IEEE Int Conf Intell Robot Syst. 2011; 1413–1419.

[25] Lui WH, Saxena A. Tangled: learning to untangle ropes with RGB-D perception. IEEE Int Conf Intell Robot Syst. 2013; 837–844.

[26] Hu Z, Sun P, Pan J. Three-dimensional deformable object manipulation using fast online Gaussian process regression. IEEE Robot Autom Lett. 2018.

[27] Jia B, Hu Z, Pan J, Manocha D. Manipulating highly deformable materials using a visual feedback dictionary. Proc IEEE Int Conf Robot Autom*.* 2018; 239–246.

[28] Corona E, Alenyà G, Gabas A, Torras C (2018). Active garment recognition and target grasping point detection using deep learning. Pattern Recognit.

[29] Sannapaneni B, Shaswat M, Nippun Kumaar AA. Learning from demonstration algorithm for cloth folding manipulator. 2017 Int Conf Adv Comput Commun Informatics, ICACCI 2017; 1393–1398.

[30] Balaguer B, Carpin S (2011). Combining imitation and reinforcement learning to fold deformable planar objects. IEEE/RSJ Int Conf Intell Robot Syst.

[31] Balaguer B. An hybrid approach for robots learning folding tasks. Learning.

[32] Kober J, Peter J. Policy search for motor primitives in robotics. In Tracts in advanced robotics. Springer, 2014.

[33] Yaqiang M, Matsubara T, Yamazaki K. Folding behavior acquisition of a shirt placed on the chest of a dual-arm robot. In: Proceedings of the 2018 IEEE International Conference on Information and Automation, ICIA 2018; 2018.

[34] Deisenroth MP, Rasmussen CE. PILCO: a model-based and data-efficient approach to policy search. In: Proceedings of the Proceedings of the 28th International Conference on Machine Learning, ICML 2011; 2011.

[35] Wu Y, Yan W, Kurutach T, Pinto L, Abbeel P. Learning to manipulate deformable objects without demonstrations. 2019.

[36] Zhang X, Ng R, Chen Q. Single image reflection separation with perceptual losses. Proc IEEE Comput Soc Conf Comput Vis Pattern Recognit. 2018; 4786–4794.

[37] Chance G, Jevtić A, Caleb-Solly P, Dogramadzi S (2017). A quantitative analysis of dressing dynamics for robotic dressing assistance. Front Robot AI.

[38] Stria J, Hlavac V. Classification of hanging garments using learned features extracted from 3D point clouds. IEEE Int Conf Intell Robot Syst. 2018; 5307–5312.

[39] Erickson Z, Clever HM, Gangaram V, Turk G, Liu CK, Kemp CC. Multidimensional capacitive sensing for robot-assisted dressing and bathing. 2019; 224–231. 10.1109/ICORR.2019.8779542 31374634

[40] Gao Y, Chang HJ, Demiris Y. Personalised assistive dressing by humanoid robots using multi-modal information. Icra. 2016.

[41] Pignat E, Calinon S (2017). Learning adaptive dressing assistance from human demonstration. Rob Auton Syst.

[42] Eddy SR (1996). Hidden Markov models. Curr Opin Struct Biol.

[43] Kapusta A, Erickson Z, Clever HM, Yu W, Liu CK, Turk G, Kemp CC (2019). Personalized collaborative plans for robot-assisted dressing via optimization and simulation. Auton Robots.

[44] Kapusta A, Yu W, Bhattacharjee T, Liu CK, Turk G, Kemp CC. Data-driven haptic perception for robot-assisted dressing. 25th IEEE Int Symp Robot Hum Interact Commun RO-MAN 2016; 451–458.

[45] Clegg A, Tan J, Karen BC, Yu W, Liu CK, Turk G (2018). 2018 learning to dress: synthesizing human dressing motion via deep reinforcement learning. ACM Trans Graph.

[46] Schulman J, Levine S, Moritz P, Jordan M, Abbeel P. Trust region policy optimization. In: Proceedings of the 32nd International Conference on Machine Learning, ICML 2015; 2015.

[47] Chance G, Camilleri A, Winstone B, Caleb-Solly P, Dogramadzi S (2016). An assistive robot to support dressing-strategies for planning and error handling. Proc IEEE RAS EMBS Int Conf Biomed Robot Biomechatr.

[48] Erickson Z, Clever HM, Turk G, Liu CK, Kemp CC. Deep haptic model predictive control for robot-assisted dressing. Proc IEEE Int Conf Robot Autom. 2018; 4437–4444.

[49] Koganti N, Shibata T, Tamei T, Ikeda K (2019). Data-efficient learning of robotic clothing assistance using Bayesian Gaussian process latent variable model. Adv Robot.

[50] Saxena K, Garment ST (2019). Garment recognition and grasping point detection for clothing assistance task using deep learning∗. IEEE/SICE Int Symp Syst Integr SII.

[51] Joshi RP, Koganti N, Shibata T. A framework for robotic clothing assistance by imitation learning. Adv Robot. 2019.

[52] Koganti N, Tamei T, Matsubara T, Shibata T (2014). Real-time estimation of Human-Cloth topological relationship using depth sensor for robotic clothing assistance. Proc IEEE Int Work Robot Hum Interact Commun..

[53] Twardon L, Ritter H (2015). Interaction skills for a coat-check robot: identifying and handling the boundary components of clothes. Proc IEEE Int Conf Robot Autom.

[54] Tamei T, Matsubara T, Rai A, Shibata T. Reinforcement learning of clothing assistance with a dual-arm robot. IEEE-RAS Int Conf Humanoid Robot. 2011; 733–738.

[55] Matsubara T, Shinohara D, Kidode M (2013). Reinforcement learning of a motor skill for wearing a T-shirt using topology coordinates. Adv Robot.

[56] Shinohara D, Matsubara T, Kidode M. Learning motor skills with non-rigid materials by reinforcement learning. 2011 IEEE Int Conf Robot Biomimetics, ROBIO 2011 2011; 2676–2681.

[57] Klee SD, Ferreira BQ, Silva R, Costeira JP, Melo FS, Veloso M. Personalized assistance for dressing users. Lect Notes Comput Sci (including Subser Lect Notes Artif Intell Lect Notes Bioinformatics) 2015; 9388 LNCS, 359–369.

[58] Canal G, Alenyà G, Torras C. Personalization framework for adaptive robotic feeding assistance.

[59] Yamazaki K (2015). A method of grasp point selection from an item of clothing using hem element relations. Adv Robot.

[60] Tsurumine Y, Cui Y, Uchibe E, Matsubara T (2019). Deep reinforcement learning with smooth policy update: application to robotic cloth manipulation. Rob Auton Syst.

[61] Gheshlaghi Azar M, Gómez V, Kappen Bkappen HJ (2012). Dynamic policy programming. J Mach Learn Res.

[62] Mnih V, Kavukcuoglu K, Silver D, Rusu AA, Veness J, Bellemare MG, Graves A, Riedmiller M, Fidjeland AK, Ostrovski G (2015). Human-level control through deep reinforcement learning. Nature.

[63] Matas J, James S, Davison AJ. Sim-to-real reinforcement learning for deformable object manipulation. arXiv Prepr. arXiv1806.07851 2018.

[64] Jiménez P (2017). Visual grasp point localization, classification and state recognition in robotic manipulation of cloth: an overview. Rob Auton Syst..

[65] Gabas, A, Corona, E, Alenyà, G, Torras, C. Robot-aided cloth classification using depth information and CNNs. Lect Notes Comput Sci (including Subser Lect Notes Artif Intell Lect Notes Bioinformatics) 2016; 9756: 16–23.

[66] Nair A, Chen D, Agrawal P, Isola P, Abbeel P, Malik J, Levine S. Combining self-supervised learning and imitation for vision-based rope manipulation. Proc IEEE Int Conf Robot Autom. 2017; 2146–2153.

[67] Kormushev P, Nenchev DN, Calinon S, Caldwell DG. Upper-body kinesthetic teaching of a free-standing humanoid robot. In: Proceedings of the Proceedings-IEEE International Conference on Robotics and Automation; 2011.

[68] Sutton RS, Barto AG. Reinforcement learning: an introduction. 2nd Edn Draft). Kybernetes 2017.

[69] Luo S, Yuan W, Adelson E, Cohn AG, Fuentes R. ViTac: feature sharing between vision and tactile sensing for cloth texture recognition. Proc IEEE Int Conf Robot Autom. 2018; 2722–2727.

[70] Tamei T, Matsubara T, Rai A, Shibata T. Reinforcement learning of clothing assistance with a dual-arm robot. In Proceedings of the Humanoid Robots (Humanoids), 2011 11th IEEE-RAS International Conference on; 2011; pp. 733–738.

[71] Clegg A, Kemp CC, Turk G, Liu CK. Modeling collaboration for robot-assisted dressing tasks. 2019.

[72] Demura S, Sano K, Nakajima W, Nagahama K, Takeshita K, Yamazaki K. Picking up one of the folded and stacked towels by a single arm robot. 2018 IEEE Int Conf Robot Biomimetics, ROBIO 2018 2019: 1551–1556.

[73] Yuan W, Mo Y, Wang S, Adelson E (2018). Active clothing material perception using tactile sensing and deep learning. IEEE Int Conf Robot Autom.

[74] Koenig N, Howard A. Design and use paradigms for Gazebo, an open-source multi-robot simulator. In: Proceedings of the 2004 IEEE/RSJ International Conference on Intelligent Robots and Systems (IROS); 2004.

[75] Michalík R, Janota A. The PyBullet module-based approach to control the collaborative YuMi robot. In: Proceedings of the 13th International Conference ELEKTRO 2020, ELEKTRO 2020-Proceedings; 2020.

[76] Fosch-Villaronga E, Grau R. Expert considerations for the regulation of assistive robotics. A European robotics forum echo. Dilemata. 2019.

[77] Clarke R. The regulation of civilian drones’ impacts on behavioural privacy. Comput Law Secur Rev. 2014.

[78] Jiménez P, Torras C. Perception of cloth in assistive robotic manipulation tasks. Nat Comput. 2020.

[79] Zhang F, Demiris Y. Learning grasping points for garment manipulation in robot-assisted dressing. Proc IEEE Int Conf Robot Autom. 2020; 9114–9120.

[80] Bazi Y, Bashmal L, Al Rahhal MM, Al Dayil R, Ajlan N. Al vision transformers for remote sensing image classification. Remote Sens*.* 2021.

[81] Ghali R, Akhloufi MA, Jmal M, Mseddi WS, Attia R. Wildfire segmentation using deep vision transformers. Remote Sens*.* 2021.

[82] Seita D, Jamali N, Laskey M, Tanwani AK, Berenstein R, Baskaran P, Iba S, Canny J, Goldberg K. Deep transfer learning of pick points on fabric for robot bed-making. 2018; 1–16.

[83] Fu Y, Jha DK, Zhang Z, Yuan Z, Ray A. Neural network-based learning from demonstration of an autonomous ground robot. Machines. 2019.

[84] Brys T, Harutyunyan A, Suay HB, Chernova S, Taylor ME, Nowé A. Reinforcement learning from demonstration through shaping. In Proceedings of the IJCAI International Joint Conference on Artificial Intelligence; 2015.

